# Recent advances progress in radiotherapy for breast cancer after breast-conserving surgery: a review

**DOI:** 10.3389/fonc.2023.1195266

**Published:** 2023-08-21

**Authors:** Yun Wang, Jingjing Shen, Peihua Gu, Zhongming Wang

**Affiliations:** ^1^ Department of Radiation Oncology, Shidong Hospital, Shidong Hospital Affiliated to University of Shanghai for Science and Technology, Shanghai, China; ^2^ School of Health Science and Engineering, University of Shanghai for Science and Technology, Shanghai, China

**Keywords:** breast cancer, breast-conserving surgery, radiotherapy, hypofractionated whole breast irradiation, accelerated partial breast irradiation

## Abstract

Adjuvant radiotherapy after breast-conserving surgery has become an integral part of the treatment of breast cancer. In recent years, the development of radiotherapy technology has made great progress in this field, including the comparison of the curative effects of various radiotherapy techniques and the performance of the segmentation times. The choice of radiotherapy technology needs to be co-determined by clinical evidence practice and evaluated for each individual patient to achieve precision radiotherapy. This article discusses the treatment effects of different radiotherapy, techniques, the risk of second cancers and short-range radiation therapy techniques after breast-conserving surgery such as hypo fractionated whole breast irradiation and accelerated partial breast irradiation. The choice of radiotherapy regimen needs to be based on the individual condition of the patient, and the general principle is to focus on the target area and reduce the irradiation of the normal tissues and organs. Short-range radiotherapy and hypofractionated are superior to conventional radiotherapy and are expected to become the mainstream treatment after breast-conserving surgery.

## 1 Introduction

According to the 2020 global cancer statistics released by the International Agency for Research on Cancer, there are 2.3 million new cases of breast cancer worldwide, overtaking lung cancer as the most common cancer, accounting for 11.7% of new cancer cases worldwide and 6.9% of mortality (1). It accounts for 24.5% of the new cases of cancer in women, and it is also the leading cause of death in women, with a mortality rate of 15.5%. Postoperative radiotherapy for breast cancer can improve the local control rate, reduce the recurrence and metastasis of breast tissue and its surrounding lymph nodes, and improve the survival rate of patients. Many studies have shown that the local control rate, distant metastasis rate and overall survival rate of breast cancer after breast conserving surgery can reach even or even better than that of traditional radical mastectomy (2–4). Therefore, it is no longer optimal to offer breast resection to patients who are suitable for breast conserving surgery (5). In order to provide reference for clinical professionals, this paper reviews the options of radiotherapy techniques and the selection of different radiotherapy protocols after breast conserving surgery.

## 2 Comparison of the effects of different radiotherapy techniques after breast conserving surgery

There are differences in the efficacy and adverse reactions of different radiotherapy techniques. In the early stage of radiotherapy, studies have shown that traditional radiotherapy technology can increase the irradiation dose of normal tissues and organs at risk (OAR), leading to related complications and reduced long-term survival rate (6). In recent years, due to the continuous progress and application of computer technology, such as three dimensional conformal radiotherapy (3D-CRT), intensity-modulated radiotherapy (IMRT),volumetric modulated arc therapy (VMAT), deep inspiration breath-hold (DIBH), helical tomotherapy (TOMO), protons, heavy ions, number of functional imaging technologies and respiratory gating technologies have improved the coverage of target areas, reduced the dose of radiation to normal tissues and OAR, and significantly improved the quality of life and overall survival rate of patients. Different radiotherapy techniques and their therapeutic effects after breast conserving surgery are described in detail below.

### 2.1 Radiotherapy techniques after breast conserving surgery

3D-CRT reconstructs the three-dimensional structure of tumors with the assistance of medical imaging images (7). Using three-dimensional treatment planning system designs several non-coplanar irregular field, through the different angle of radiation field and the tumor shape similar to block selection, from three-dimensional direction to irradiate of target area, makes the high dose area on the shape of the dose distribution in three-dimensional direction is consistent with the target shape, maximizes the killing of tumor cells while reducing the radiation on the surrounding normal tissues to a large extent. IMRT was further developed on the basis of 3D-CRT (8). Compared with 3D-CRT, IMRT uses multiple irradiation fields to optimize the dose distribution of the target area, resulting in a more uniform distribution of the high-dose area and a steeper dose gradient at the edge of the planned target area. IMRT can also achieve different dose levels within a plan to meet the dose needs of different target areas. With the rapid development of hardware and software in the field of radiotherapy, VMAT emerged by integrating the fixed field IMRT and pull arc irradiation (9). VMAT rotary irradiation has a wide angle and a more uniform dose distribution, which can improve the conformity of the tumor target area and reduce the relative treatment time. However, it generally requires more sophisticated inverse dose optimization design software and dose verification system. There are two types of image-guided radiotherapy (IGRT) based on imaging guidance and breath-control technology. The imaging techniques used in the former include electronic portal imaging device (EPID), cone beam computed tomography (CBCT), 4D-CT and magnetic resonance imaging (MRI). The latter includes deep inspiration breath-hold (DIBH), Respiratory gating technology and so on (10, 11). IGRT combines medical accelerator with imaging equipment, and at the same time considers the errors caused by positioning and patient breathing movement in the process of radiotherapy. Therefore, being able to monitor radiotherapy in real time, it improves the accuracy of tumor irradiation, as well as the protection the surrounding normal organs and tissues. TOMO is a radiotherapy technology integrating intensity modulated radiotherapy, image-guided and many other technologies (12). In the process of treatment, the radiation can rotate 360° around the target area, patients are moved through the gantry bore simultaneously. In this case, doses were delivered from any angle transmission to the tumor, thus improving the target high uniformity and highly conformal dose distribution, and synchronous observation to the target area and the change of the normal tissue, further enabling timely revision of the radiotherapy plan, and achieving adaptive radiation.

Compared with photon ray therapy, the advantages of proton and heavy ion therapy are mainly based on their physical dose distribution. Most of their energy is deposited at the tissue depth (Bragg peak), reducing the overall dose received by normal tissues. Heavy ions have high linear energy transfer (LET) properties and are considered to have biological advantages, including higher cell-killing efficiency. At present, the heavy ions used in clinical radiotherapy are mainly carbon ions. The dose deposition of carbon ion beam is low in the entrance area of normal tissue exposure, but greatly enhanced at the end of tumor range, and then suddenly decreased to close to zero, which can achieve “targeted explosion” of tumor. Dosimetric comparative analyses have identified the advantages of proton and carbon ion therapy for better heart protection with radiotherapy to chest wall ± breast target (13, 14).

### 2.2 Whole breast irradiation

Liu et al. compared the dosimetry differences of the three radiotherapy techniques after left breast conserving surgery (BCS). 3D-CRT, IMRT and VMAT all met the clinical dose requirements. Except for homogeneity index (HI), all the dose evaluation parameters of 3D-CRT were inferior to IMRT and VMAT. IMRT and VMAT have better target consistency, VMAT can effectively reduce the number of Monitor Units and radiotherapy time, only 49.33% and 55.86% of those of IMRT, respectively (9). As the technique continued to, the advantages of 3D-CRT and IMRT were combined to create a new radiation therapy technique: Hybrid IMRT. Previous studies showed that the Hybrid IMRT composed of a pair of tangent field conformal plan and four field intensity regulation plan had better uniformity of dose distribution than the general IMRT, was more uniform than general IMRT, and the volume of low-dose irradiation area was reduced. In the case that the positioning accuracy is not guaranteed, Hybrid IMRT is a better choice (15). Mo et al. analyzed the dosimetry of VMAT, step-shoot IMRT and 3D-CRT for left breast cancer after BCS, and found that VMAT had better conformity compared with step-shoot IMRT and 3D-CRT. The volume of high-dose radiation was smaller in the ipsilateral lung and heart, but the VMAT had a higher volume of low-dose radiation in the contralateral lung (16). Studies have pointed out that VMAT combined with DIBH technology can effectively reduce the low-dose region (17). Compared with VMAT with partial arcs (pVMAT), tangential IMRT which use tangential field and 3D-CRT combined with DIBH are better technologies than VMAT with Partial arcs, and could protect heart tissue and another OAR without affecting target coverage of early breast cancer irradiation (18). Xie et al. studied seven whole-breast radiotherapy techniques after left BCS, dosimetric comparisons and risk assessments were performed for conventional tangential, field-in-field (FIF), Hybrid IMRT, IMRT, standard VMAT, noncoplanar VMAT (NC-VMAT), and Multiple arc VMAT (MA-VMAT) (19). FIF was the main field with two parallel opposed fields at an angle to each other, and each main field is set with 2-3 sub-fields to achieve the purpose of dose optimization. Hybrid IMRT plans included a pair of open tangent fields and a pair of dynamic IMRT tangent fields, and 80% of prescription dose was delivered by open tangent beams and 20% of the prescription dose was delivered by IMRT beams. NC-VMAT is a radiotherapy technology that used multiple non-coplanar arcs whose formation trajectories are not in the same plane for irradiation. In the treatment process, not only the gantry can be rotated, the treatment bed and multi-leaf collimator (MLC) could also be rotated to achieve 360° rotation to treat patients, with high target fitness and faster treatment speed (20). STD-VMAT utilized two coplanar partial arcs, and the MA-VMAT plan consisted of six partial arcs. The results showed that NC-VMAT and MA-VMAT were the preferred radiotherapy techniques for breast cancer patients after BCS. Furthermore, the dose of OAR in the five inverse planning techniques were lower than the two forward planning techniques. NC-VMAT used for the first time in total breast radiotherapy for breast cancer had the highest target conformity and the lowest cardiopulmonary dose. MA-VMAT had the optimal dose of organ at risk and the lowest risk of late side effects.

A novel internal technique “Non-Uniform VMAT (NU-VMAT) “ has been developed for automatic reduction of cardiac dose and optimization of treatment plan in left breast radiotherapy in China. In traditional VMAT, the sub-fields were evenly distributed at 360°, which may bring dose radiation to normal tissues in some directions. The NU-VMAT model that is developed through its internal code proposed in this study can eliminating unnecessary beams based on traditional VMAT and increase the radiation intensity of the remaining significant fields. In addition, NU-VMAT can properly adjust the rotation speed of the gantry and provide two treatment modes. At the circumstance of low-speed gantry rotation matching fast MLC motion, multiple sub-fields radiations can be completed by the rapid MLC movement, or the modulation intensity can be increased by extending partial arc. On the contrary, when high-speed gantry rotation matches static MLC, large-field radiation can be completed while creating multiple beam-off fields simultaneously. The results showed that NU-VMAT achieved better dose control with the mean cardiac dose declined by 32.2% compared with VMAT and 4.4% compared with IMRT, effectively reducing cardiotoxicity. Moreover, compared with IMRT, the Monitor Units of NU-VMAT was reduced by 69.8%, and the delivery time of NU-VMAT declined by 28.4%, which greatly reduced the treatment time of patients (21). NU-VMAT was conducive to the control of patients’ activities during radiotherapy and the increase of patients’ compliance (**Table 1**).

**Table 1:** Dosimetry comparison of radiotherapy techniques in some of the trials in this review.

Author (reference)	>HI( $\bar{\text{x}}$ S)	CI( $\bar{\text{x}}$ S)	D_mean_( $\bar{\text{x}}$ S, cGy)				MU( $\bar{\text{x}}$ S)
			Heart	Ipsilateral lung	Contralateral lung	Contralateral breast	
Liu et al. (9)	3D-CRT: 1.12 ± 0.11 IMRT: 1.10 ± 0.09 VMAT: 1.10 ± 0.10	3D-CRT: 0.33 ± 0.08 IMRT: 0.77 ± 0.05 VMAT: 0.79 ± 0.05	3D-CRT: 881.53 ± 51.56 IMRT:1302.42 ± 67.22 VMAT:1367.39 ± 61.64	3D-CRT: 1385.14 ± 110.34 IMRT: 1353.21 ± 98.43 VMAT: 1378.37 ± 83.42	3D-CRT: 107.77 ± 18.23 IMRT: 466.22 ± 34.45 VMAT: 532.18 ± 42.31	3D-CRT: 624.71 ± 50.96 IMRT: 667.57 ± 64.81 VMAT: 844.65 ± 57.29	3D-CRT:496 ± 27 IMRT:827 ± 31 VMAT:408 ± 16
Ouyang et al. (15)	IMRT: 1.09 ± 0.02 Hybrid IMRT: 1.07 ± 0.01	IMRT: 0.68 ± 0.10 Hybrid IMRT: 0.66 ± 1.42	NA	IMRT: 1129.09 ± 76.65 Hybrid IMRT: 1112.01 ± 61.47	IMRT: 139.75 ± 58.59 Hybrid IMRT: 173.76 ± 40.09	IMRT: 454.39 ± 274.80 Hybrid IMRT: 369.02 ± 187.20	IMRT:699.25 ± 79.38 Hybrid IMRT: 553.50 ± 92.53
Mo et al. (16)	VMAT: 0.10 ± 0.01 sIMRT: 0.09 ± 0.01 3D-CRT: 0.13 ± 0.01	VMAT: 0.77 ± 0.03 sIMRT: 0.68 ± 0.02 3D-CRT: 0.55 ± 0.01	VMAT: 496.74 ± 134.94 sIMRT: 592.27 ± 201.74 3D-CRT:954.61 ± 332.64	VMAT: 838.58 ± 113.62 sIMRT: 987.91 ± 152.67 3D-CRT: 1392.44 ± 183.70	VMAT: 185.10 ± 22.59 sIMRT: 110.98 ± 17.51 3D-CRT: 35.81 ± 1.71	VMAT: 355.64 ± 38.97 sIMRT: 211.64 ± 54.60 3D-CRT: 69.54 ± 40.38	VMAT: 875.0 ± 124.4 sIMRT: 486.7 ± 85.5 3D-CRT: 419.8 ± 49.9
Vikström et al. (18)	3D-CRT(FB):0.11 ± 0.01 3D-CRT (DIBH):0.11 ± 0.01 tIMRT:(DIBH):0.10 ± 0.01 pVMAT:(DIBH): 0.10 ± 0.01	3D-CRT(FB):0.69 ± 0.04 3D-CRT (DIBH):0.72 ± 0.05 tIMRT:(DIBH):0.80 ± 0.05 pVMAT:(DIBH):0.90 ± 0.03	3D-CRT(FB):620 ± 440 3D-CRT (DIBH):130 ± 110 tIMRT:(DIBH):110 ± 60.00 pVMAT:(DIBH):160 ± 100	3D-CRT(FB):680 ± 120 3D-CRT (DIBH):630 ± 110 tIMRT:(DIBH):570 ± 100 pVMAT:(DIBH):700 ± 110	NA	3D-CRT(FB):40 ± 30 3D-CRT (DIBH):40 ± 30 tIMRT:(DIBH):30 ± 40 pVMAT:(DIBH):250 ± 130	3D-CRT(FB):197 ± 6 3D-CRT (DIBH):198 ± 8 tIMRT:(DIBH):310 ± 63 pVMAT:(DIBH):342 ± 41
Xie et al. (19)	SOC: 0.150 ± 0.033 FIF: 0.132 ± 0.021 Hybrid IMRT: 0.126 ± 0.024 IMRT: 0.142 ± 0.029 STD-VMAT: 0.157 ± 0.056 NC-VMAT: 0.158 ± 0.044 MA-VMAT: 0.164 ± 0.046	SOC: 0.5 ± 0.1 FIF: 0.5 ± 0.1 Hybrid IMRT: 0.6 ± 0.1 IMRT: 0.8 ± 0.1 STD-VMAT: 0.8 ± 0.1 NC-VMAT: 0.8 ± 0.1 MA-VMAT: 0.8 ± 0.1	SOC:960 ± 370 FIF: 810 ± 370 Hybrid IMRT: 810 ± 280 IMRT: 740 ± 130 STD-VMAT:780 ± 150 NC-VMAT: 580 ± 100 MA-VMAT: 550 ± 120	NA	NA	SOC:280 ± 250 FIF: 190 ± 180 Hybrid IMRT: 160 ± 120 IMRT: 140 ± 70 STD-VMAT:110 ± 30 NC-VMAT: 120 ± 70 MA-VMAT: 120 ± 40	SOC:7343 ± 962 FIF: 5647 ± 227 Hybrid IMRT: 8673 ± 1506 IMRT: 19300 ± 2471 STD-VMAT:10263 ± 1049 NC-VMAT: 10397 ± 1549 MA-VMAT: 11523 ± 1167
Qiu et al. (21)	NA	IMRT:1.22 VMAT: 1.24 NU-VMAT: 1.21	IMRT: 563 ± 61 VMAT:794 ± 52 NU-VMAT: 538 ± 46	IMRT: 1169 ± 53 VMAT:1685 ± 42 NU-VMAT: 1477 ± 39	NA	NA	IMRT: 864.4 ± 171.2 VMAT:245.7 ± 11.1 NU-VMAT: 261.6 ± 41.5

HI, homogeneity index; CI, conformity index; D_mean_, mean does; MU, monitor units;3D-CRT, three dimensional conformal radiotherapy; IMRT, intensity-modulated radiotherapy; VAMT, volumetric modulated arc therapy; NA, not applicable; sIMRT, step-shoot intensity modulated radiation therapy; FB, free breathing; DIBH, deep inspiration breath hold; tIMRT, tangential IMRT; pVMAT, VMAT with partial arcs; SOC, standard of care; FIF, field-in-field; STD-VMAT, standard-VMAT; NC-VMAT, non-coplanar-VMAT; MA-VMAT, multiple arc VMAT; NU-VMAT, non-uniform VMAT.

Due to the anatomic structure of some patients, the radiation dose to OAR, such as the heart is still relatively high even with advanced photon radiation techniques. In theory, protons therapy can deposit dose to a target volume with great precision while better protecting surrounding normal tissue. Several studies have confirmed that protons therapy in breast cancer reduces the exposure of normal tissues (22, 23). In the actual treatment process, Bragg peak release will cause great damage to the surrounding normal tissues if it is not focused to the specific location of the tumor or slightly offset. Zhang et al. proved that the accuracy of protons radiotherapy in their institution reached millimeter level by establishing a quantitative evaluation method, which filled the gap in domestic research in this field and laid a foundation for the subsequent evaluation of the accuracy of heavy ion radiotherapy (24). Currently, there is a lack of clinical data on the use of carbon ions in the treatment of breast cancer. To date, there have been no large clinical trials using carbon ions radiotherapy in the treatment of breast cancer (25). Karasawa et al. designed a dose-increasing study for 7 patients with low-risk stage I breast cancer who met the requirements, giving drugs in 4 times within one week, with 3 patients receiving 48Gy, 3 patients receiving 52.8Gy, and 1 patient receiving 60Gy. Grade 1 acute skin reaction occurred in 4 patients without other adverse effects. Unfortunately, the researchers found not enough treatment effect after 3 months of treatment (26). However, subsequent studies by Karasawa et al. showed that assessment three months after carbon ions therapy was considered premature. Although the time needed from carbon ions radiotherapy to tumor disappearance was longer than expected, carbon ions therapy was considered effective and safe in stage I breast cancer patients (27).

### 2.3 Whole breast combined with regional nodal irradiation

Breast cancer can spread to regional lymph nodes, primarily including the axillary, internal mammary and supraclavicular lymph nodes. Regional nodal irradiation (RNI) also known as Regional nodal irradiation, which is commonly used for high-risk early-stage and locally advanced breast cancer patients in order to reduce locoregional recurrence(LR) and distant metastasis to improve overall survival (OS). Several studies have shown that adding RNI on the basis of WBI after BCS was beneficial to reduce the risk of distant recurrence and mortality of breast cancer (28, 29).

MA.20 trial conducted a randomized trial for patients with node-positive or high-risk node-negative. All patients were randomly assigned with whole breast irradiation plus regional nodal irradiation (including internal mammary, supraclavicular and axillary lymph nodes) or whole breast irradiation (control group). The median follow-up time was 9.5 years, there was no significant difference in survival rate between the nodal-irradiation group and the control group, with a rate of 82.8% in the nodal-irradiation group and 81.8% in the control group, and disease-free survival (DFS) rates were 82.0% and 77.0% respectively (28). In patients with node-positive or high-risk node-negative, although the increase of RNI on the basis of WBI did not increase OS, it reduced the recurrence rate of breast cancer. The EORTC trial was a randomized, phase 3 trial involving 4004 patients from 46 Institutions in 13 countries. All patients were randomly divided into two groups: the whole breast or chest wall plus RNI (nodal-irradiation group) and the control group only receiving whole breast or thoracic wall irradiation. The regional lymph nodes for irradiation were internal mammary, medial supraclavicular lymph nodes and undissected part of the axilla (29). Results of 10-year follow-up, the DFS rate was 72.1% in the nodal-irradiation and 69.1% in the control group; the distant DFS rate was 78.0% in the nodal-irradiation group and 75.0% in the control group, and OS did not differ significantly, 82.3% in the nodal-irradiation group and 80.7%, in the control group. The results showed that RNI increased the DFS rate and distant DFS rate of breast cancer, and reduced the mortality rate of breast cancer, but had little effect on the overall survival rate. 15-year results of EORTC trial also showed that in stage I–III breast cancer, internal mammary and medial supraclavicular irradiation significantly reduced breast cancer mortality and recurrence rate. However, this is not converted to improve OS (30).

Patients after breast cancer surgery with node-positive usually received ipsilateral chest wall or breast plus RNI. Takano et al. found that compared with 3D-CRT and TomoHelical, TomoDirect could provide better target dose distribution and dose retention in normal organs (31). Xu et al. applied TOMO, VMAT, IMRT and Hybrid IMRT to the radiotherapy of the whole breast, internal mammary and supraclavicular lymph nodes after BCS on the left side (32). The conformal index (CI) of TOMO, VMAT, IMRT and Hybrid IMRT were 0.83, 0.82, 0.80 and 0.77 (*P <*0.001), and HI of TOMO, VMAT, IMRT and Hybrid IMRT were 1.07, 1.11, 1.14, 1.14 (*P <*0.001). TOMO and VMAT had higher conformation and uniform target coverage, better protecting OAR, and the overall effect were significantly better than IMRT and Hybrid IMRT. TOMO usually has a longer treatment time, but TOMO is often the first choice without considering the duration of treatment. If dose distribution, treatment time, and availability matter, VMAT should be chosen. Moreover, another trial also proved that VMAT was a safe and feasible technique for irradiation of whole breast combined RNI after BCS (33).

With the development of technology, internal mammary nodes (IMN) are increasingly included in radiotherapy for early breast cancer. A study found that inverse-planned multibeam IMRT was dosimetrically feasible for whole breast plus regional lymph nodes including IMN in high-risk breast cancer patients. At 5 years, the rate of LRR, DFS and OS were 93.2%, 63.6% and 80.3% respectively. Although the low-dose area of lung was large, the incidence of Grade 3 radiation pneumonitis was very low, only 0.96% (34). An innovative design of the complete-directional-complete block (CDCB) technique for TOMO can reduce the low-dose region of traditional TOMO. Complete-block (CB), a rectangular structure with ends connected at the edge of planning target volumes (PTV), was designed to prevent the beamlets from passing through the structure. The directional blocking area of CDCB was determined by the intersection of the CB and beam aperture through the 0.5cm margin of the PTV of the IMN. If the blocked structure was proximal to the target, the directional block was used to close the beamlets to limit the beamlet entrance direction. There was also organ-based directional block (OBDB), which limited the main beam from entering the heart, lungs and contralateral breast. The use of TOMO with CDCB technology in patients with left breast cancer, including RNI and IMN irradiation areas, not only achieved better volume coverage, homogeneity and dose conformity consistency, but also better protection of the heart and bilateral lungs, and reduced radiation dose to the heart and lung (35). In another study, proton radiotherapy for IMN showed better toxicity and disease control rates at a median follow-up of 55 months than conventional radiotherapy. The five-year local regional failure rate and OS were 1.5% and 91%, and only one patient developed grade 2 radiation pneumonitis (36). Radiotherapy of IMN usually leads to higher doses to the heart and lungs. Research showed that per gray increase in the mean dose to the heart would increase the incidence of heart disease and coronary events by 7.4%, and the long-term survival rate would be reduced. Therefore, it is particularly important to reduce the irradiation to the heart and lungs during radiotherapy (37). DIBH technology is a motion management technology that can effectively reduce the dose to heart during radiotherapy. It can distinguish the heart from the target in physical structure. Ranger et al. compared the dosimetry of 3D-CRT, VMAT and proton therapy in IMN irradiation. Under the condition of autonomous deep inhalation breath-hold, 3D-CRT can achieve better IMN coverage while meeting the cardiopulmonary dose limit. However, VMAT is still considered the best photon radiotherapy technology, and the dose of OAR of proton radiotherapy is the lowest (38). When the target region contains IMN, Proton-DIBH had more advantages in cardiopulmonary protection than Photon-DIBH, with an average cardiac dose of 0.23gy and 1.19gy. Even without the use of DIBH technology, the toxicity of proton radiotherapy to OAR such as heart is still low compared with photon radiotherapy (39).

IMN as the first station of lymphatic drainage of breast cancer plays an important role in the prognosis of breast cancer. Although several trials have demonstrated the benefit of RNI for patients, given both the EORTC and MA2.0 trials targeted both the internal mammary and the supraclavicular, it is very hard to determine what proportion of the RNI to DFS was due to irradiation to the internal mammary. A randomized trial in France found that IMN irradiation did not improve OS or DFS (40). However, another prospective trial found that IMN irradiation increased 8-year OS from 72.2% to 75.9% (*P* =0.005) (41). In the context of modern systemic therapy, a retrospective analysis also found that IMN irradiation significantly improved patient DFS from 64.9% to 75.9% (*P <*0.001) (42). IMN irradiation remains controversial internationally, and more clinical studies are needed in the future to verify whether IMN irradiation is truly beneficial (**Table 2**).

**Table 2:** Comparison of clinical studies on whole breast combined with regional lymph node irradiation in this review.

Authors (reference)	Number of patients	Study design	Radiation coverage of regional lymph nodes	Proportion of patients with positive lymph nodes	Median F/U (years)	main findings		
						10 years OS	10 years DFS	AE
Whelan et al. (28)	1832	WBI+RNI VS. WBI	IM, supraclavicular and axillary lymph nodes	90%	9.5	82.8% VS. 81.8%(*P* =0.38)	82.0% VS. 77.0% (*P* =0.01)	≥2 grade acute pneumonitis 1.2%VS.0.2%(*P* =0.01) and Lymphedema 8.4%VS.4.5%(*P* =0.001)
Poortmans et al. (29, 30)	4004	IM-MS irradiation VS. no IM-MS irradiation	IM-MS lymph nodes and undissected part of the axilla	56%	15.7	15 years:73.1% VS. 70.9% (*P* =0.36)	15 years:60.8% VS. 59.9% (*P* =0.18)	Any grade of pulmonary fibrosis 5.1% VS. 2.3% Any cardiac disease 8.6% VS. 7.2%
Hennequin et al. (40)	1407	IM chain irradiation VS. IM chain no-irradiation	IM chain	NA	8.6(whole population) 11.3(survivors)	62.6% VS. 59.3% (*P* =0.8)	53.2% VS. 49.9% (*P* =0.35)	Late side effects of radiation were roughly same in two groups
Thorsen et al. (41)	3089	IMNI VS. no IMNI	IM node	100%	8.9	8 years:75.9% VS.72.2% (*P* =0.05)	NA	NA

F/U, follow-up; OS, overall survival; DFS, disease-free survival; AE, Adverse events; WBI, whole breast irradiation; RNI, Regional nodal irradiation; IM-MS, internal mammary-medial supraclavicular; IM, internal mammary; IMNI, internal mammary node irradiation; NA, not applicable.

## 3 Second cancer risk caused by different radiotherapy techniques after breast-conserving surgery for breast cancer

The current prognosis for breast cancer patients is better and the expected survival is longer, so it is especially important to reduce second cancer risk after BCS for breast cancer. Grantzau et al. conducted a meta-analysis on second non-breast cancer after radiotherapy for breast cancer and found that radiotherapy for breast cancer was significantly associated with increased risk of second non-breast cancer (43). With the increase of the number of MU and irradiation time, the patient is exposed to more leakage radiation and increased risk of systemic radiation. These leakage doses increase the risk of late side effects in the patient’s off-target organs and secondary cancers as patients live longer. The irradiation time also causes an increase in overall treatment time, excessive treatment time will lead to the reduction of radiobiological effect and the reduction of radiation accuracy caused by the change of patient position. Therefore, selecting tailored radiotherapy techniques for each patient to reduce the number of MU and the irradiation time can prevent the surrounding normal tissues from more leakage dose radiation, reduce the risk of secondary cancer and improve the survival benefit of patients (9, 44, 45). Han et al. compared the risk of second cancer caused by five different radiotherapy techniques (3DCRT, IMRT, VMAT, FIF and TOMO) during whole breast irradiation (46). TOMO was found to provide plan quality comparable to IMRT with the lowest lifetime attributive risk (LAR) for adjacent organs. So it may be a better treatment for younger patients with a longer expected survival. Compared with tangential VMAT (t-VMAT), t-IMRT significantly reduced the dose of OAR and the overall dose of normal tissues, especially reduced the risk of second cancer in contralateral lung and contralateral breast (47).

In theory, proton therapy can irradiate a target volume with great precision, while better protect surrounding normal tissue. Some studies have found that proton therapy can reduce the risk of second cancer in left breast cancer radiotherapy compared with mixed IMRT (48). According to modelling studies, proton radiotherapy reduced the overall dose by two to three times compared to photon-based treatments, and may reduce the risk of secondary cancer by 2 to 15 times (49). However, clinical validation has been lacking due to limited access to follow-up and use of proton radiotherapy. In the future, with the continuous development of proton technology and screening of suitable patients, this can be further verified.

## 4 Short-range radiation therapy techniques after breast-conserving surgery

Different from conventional fractionated radiotherapy, short-range radiotherapy after BCS includes hypofractionated whole breast irradiation and accelerated partial breast irradiation. Both can effectively shorten the treatment time and reduce the treatment cost. The clinical efficacy of the two radiotherapy modes will be discussed below.

### 4.1 Hypofractionated whole breast irradiation after BCS

The conventional fractionated of postoperative radiotherapy for breast cancer is a dose of 45.0Gy ~ 50.4Gy/25 ~ 28 fractions over 5 weeks. Boost irradiation (BI) was added to the tumor bed with a total dose of 10 to 16Gy was delivered in 4 to 8 fractions of 2Gy or 2.5Gy (50). Conventional fractionation takes longer and costs more, and patients are often unable to complete the entire radiotherapy plan.

The segmentation method of radiation dose mainly depends on the α/βvalue of tumor tissue. Radiobiology studies based on tumors have found that hypofractionated therapy can achieve good results in the treatment of breast cancer (51). Haviland et al. reported the results of the 10-year follow-up of the UK START trial. In the START-A, 2236 patients were randomly divided into three groups and a regimen of 50Gy in 25 fractions over 5 weeks was compared with 41.6Gy or 39Gy in 13 fractions over 5 weeks. Median follow-up was 9.3 years. In START-B, 2,215 patients were randomly divided into two groups, and a regimen of 50Gy in 25 fractions over 5 weeks was compared with 40Gy in 15 fractions over 3 weeks. Median follow-up was 9.9 years. The results showed that compared with conventional fractionation, there was no significant difference in local recurrence rate in 10 years, and the incidence of breast edema, telangiectasia and other adverse reactions were lower. hypofractionation with appropriate dose was safe and effective for patients with early breast cancer (52). Cao et al. divided 176 patients with early breast cancer into two groups. In the hypofractionated group, 106 patients received 16 fractions of 2.66Gy for the total breast following 5 fractions of 2Gy for local tumor bed. In conventional group, 70 patients received 25 fractions of 2Gy for the total breast following 5 fractions of 2Gy for local tumor bed. The 3-year local control rates were 97.1% and 94.3% in the hypofractionated and conventional fractionation groups, with no statistically significant differences (53). Nozaki et al. adopted the same hypofractionated dose fractions, and BI of 10.64Gy in 4 fractions was added when the surgical margin was ≤5mm. The median follow-up was 70.5 months. The proportion of late adverse reactions was 4.3%, and 5-year OS, DFS and ipsilateral breast relapse-free survival were 98.7%, 95.4% and 98.0%, respectively. The results showed that for Asian women with margin-negative invasive breast cancer after BCS, HF-WBI offered satisfactory local control with acceptable late adverse effects and good cosmetic outcomes (54). Wang et al. researched 734 patients with T_1-2_N_0-3_ invasive breast cancer after BCS to determine whether the 3.5-week fractionated radiotherapy plan in China was the same as the standard 6-week radiotherapy plan. Conventional fractionation group(n=366) with a total dose of 50Gy in 25 fractions over 5 weeks and the BI was added to the tumor bed with a dose of 10Gy in 5 fractions. Hypofractionated group(n=368) with a total dose of 43.5Gy in 15 fractions over 3 weeks and the tumor bed with a daily dose of 8.7Gy in 3 daily fractions. The median follow-up time was 73.5 months. It was found that the cumulative incidence of 5-year local recurrence was 1.2% in the hypofractionated radiotherapy group and 2.0% in the conventional fractionation radiotherapy group. As far as LR is concerned, hypofractionated radiotherapy may not be inferior to conventional fractionated radiotherapy. Hypofractionated radiotherapy is at least as effective as conventional fractionation radiotherapy in local control, survival and late adverse reactions (55). The FAST-Forward trial compared the 5-year efficacy of a one-week plan of hypofractionated radiotherapy with a three-week plan. 4096 patients were divided into three groups. A dose of 40Gy in 15 fractions over 3 weeks, a dose of 27Gy in 5 fractions over 1 week, and a dose of 26Gy in 5 fractions over 1 week, the median follow-up time was 71.5 months. In the 40Gy, 27Gy and 26Gy groups, the proportion of moderate or severe normal tissue effect in breast or chest wall was 9.9%, 15.4% and 11.9%, respectively. Normal tissue at 27Gy group had higher risk. For patients with early breast cancer who received adjuvant local radiotherapy after the first operation, the local tumor control rate of 26Gy in 5 fractions within 1 week was no less than the of 40Gy in15 fractions within 3 weeks, and the normal tissue effect was also safe within 5 years (56).

The ASTRO has made 40Gy in15 fractions or 42.5Gy in16 fractions, 5 times a week, as the first choice for the women with invasive breast cancer receiving whole breast radiotherapy after breast cancer surgery with or without inclusion of low axilla (57). In 2021, Guidelines for Diagnosis and Treatment of Breast Cancer of Chinese Anti-Cancer Association recommended that the hypofractionated radiotherapy was 40 to 42.5Gy in 15 to 16 fractions, 2.66Gy in 1 fraction, 5 fractions per week or adopted the segmentation of the Cancer Hospital of the Chinese Academy of Medical Sciences, and it was 43.5Gy in15 fractions, 2.9Gy in 1fraction, 5 fractions per week (50, 55). Hypofractionated radiotherapy can reduce the treatment period and cost of radiotherapy for breast cancer and improve the quality of life of patients (**Table 3**).

**Table 3:** Clinical trials of hypofractionated whole breast irradiation in this review.

Authors (reference)	Number of patients	Country/stage	Fractionation schedule TTD/fractions/OTT	Median F/U (months)	Results
Haviland et al. (52)	START A: 2236 START B: 2215	UK	START A: 50Gy/25 fractions/5 weeks 41.6Gy/13 fractions/5 weeks 39Gy/13 fractions/5 weeks START B: 50Gy/25 fractions/5 weeks 40Gy/15 fractions/3weeks	START A: 9.3 years START B: 9.9 years	10 years local-regional relapse: START A: 41.6Gy group VS. 50Gy group (6.3% VS. 7.4%, HR 0.91; *P* =0.65) 39Gy group VS. 50Gy group (8.8% VS. 7.4%, HR 1.18; *P* =0.41) START B: 40Gy group VS. 50Gy group (4.3% VS. 5.5%, HR 0.77; *P* =0.21)
Cao et al. (53)	176	China	42.56Gy/16 fractions/NA 50Gy/25 fractions/5 weeks	27	The 3 years local control rate: HF VS. CF (97.1% VS. 94.3%, *P >*0.05)
Nozaki et al. (54)	306	Japan	42.56Gy/16 fractions/NA	70.5	5 years OS, DFS were 98.7%, 95.4% respectively.
Wang et al. (55)	734	China	50Gy/25 fractions/5 weeks 43.5Gy/15 fractions/3 weeks	73.5	The 5-year incidence of local recurrence: HF VS. CF (1.2% VS. 2.0%, HR 0.62; *P* =0.017)
Brunt et al. (56)	4096	UK	40Gy/15 fractions/3 weeks 27Gy/5 fractions/1 week 26Gy/5 fractions/1 week	71.5	The ipsilateral breast tumor relapse: 40Gy VS. 27Gy VS. 26Gy (2.3% VS.2.0% HR 0.86 VS.1.5% HR 0.67)

TTD, total tumor dose; OTT, overall treatment time; F/U, follow-up; NA, not applicable; HF, hypofractionated; CF, conventional fractionation; HR, hazard ratio; OS, overall survival; DFS, disease-free survival.

### 4.2 Accelerated partial breast irradiation after BCS

APBI refer to radiation at the tumor bed and surrounding high-risk recurrence areas, which reduces the radiation range compared with whole breast radiation (WBI). APBI includes brachytherapy and external beam radiotherapy, the brachytherapy mainly includes multicatheter interstitial brachytherapy, balloon-based brachytherapy, and intraoperative radiation therapy(IORT) (58). The external beam radiotherapy mainly includes 3D-CRT and IMRT. NCCN guidelines recommend the APBI radiotherapy plan as brachytherapy 34Gy in 10 fractions, twice a day; External beam radiotherapy 38.5Gy in 10 fractions, twice a day, improved the single dose, shortened the radiotherapy time to one week (59).

Strnad et al. randomly divided 1184 screened patients after BCS into WBI group (n=551) and APBI group (n=633). In the WBI group, 50.0 to 50.4Gy in 25 to 28 fractions, tumor bed with a dose of 10.0Gy in 5fractions; The APBI group received brachytherapy with multicatheter (60). The results showed that the 5-year local control rate, DFS, OS and skin adverse reactions of brachytherapy with multicatheter after breast-conserving surgery for early breast cancer were similar to those of WBI. In the IMPORTLOW trial, 2018 patients after BCS were randomly divided into three groups: whole breast 40Gy radiotherapy group, whole breast 36Gy radiotherapy plus tumor-bed 40Gy radiotherapy reduction group, and local 40Gy radiotherapy group, with a median follow-up of 72.2 months (61). The local recurrence rate was 1% in the whole breast radiotherapy group, 0.2% in the dose reduction group and 0.5% in the APBI group. The local recurrence rate of the APBI and the dose reduction group was lower than that of the whole breast radiotherapy group, and the adverse reactions were significantly reduced. However, Olivotto et al. previously showed that 38.5Gy in 10 fractions, twice daily 3D-CRT accelerated partial breast radiotherapy increased the incidence of late radiation toxicity and produced poor cosmetic effects compared with standard whole breast radiotherapy (62). Long-term follow-up results showed that the APBI group had a worsening trend of cosmetic effects. The incidence of adverse cosmetic effects assessed by nurses at 3, 5 and 7 years was 29%, 32% and 36%, respectively. However, APBI was not inferior to WBI in the prevention of ipsilateral breast tumor recurrence (IBTR), with an 8-year cumulative IBTR rate of 3.0% and 2.8% in the two groups, respectively (63).The NSABP/RTOG phase 3 trial was a large trial with a total of 4216 patients from 154 clinical centers in the United States, Canada, and Ireland et al. APBI group (n=2107): brachytherapy with a total dose of 34Gy in10 fractions, twice daily with at least 6 hours interval or 3DCRT with a total dose of 38.5Gy in 10 fractions twice daily with at least 6 hours interval; WBI group (n=2109): the dose of 50Gy in 25 fractions. The median follow-up of 10.2 years. APBI and WBI IBTR 10 years cumulative incidence of 4.6%, 3.9% respectively, the absolute difference is less than 1%, The risk of secondary cancers and treatment-related toxicities were similar between the two groups, and APBI was an acceptable alternative for some women (64). However, the study was unable to demonstrate non-inferior of APBI due to the inclusion of high-risk node-positive patients or the use of multiple APBI techniques. Based on these trials, twice-daily 3D-CRT APBI was not recommended for routine treatment.

Compared with the APBI using 3DCRT, the use of IMRT for external irradiation treatment can make the dose distribution more uniform, reduce the occurrence of adverse reactions and improve the cosmetic effect. Livi et al. used IMRT for partial breast irradiation, and the same treatment plan of APBI was 30Gy in 5 fractions, the WBI plan was 50Gy in 25 fractions. 5-year follow-up showed no significant difference in IBTR between the two groups, and APBI showed better cosmetic results with significantly reduced acute and late skin toxicity (65). Results showed at a median follow-up of 10.7 years that the IBTR and were 2.5% in WBI group and 3.7% in APBI group, showing no significant difference. APBI showed lower toxicity associated with acute and late treatment and better cosmetic results (66). A meta-analysis of the safety and efficacy of APBI and WBI in early breast cancer showed that APBI had no significant difference with WBI in DFS, OS, adverse reactions and cosmetic effects. APBI reduced acute skin reactions, but had a high local recurrence rate (67). Based on these trials, patients receiving APBI should be carefully screened, and IMRT should be preferred for external beam radiotherapy.

IORT is a technology that uses electron beam or low-energy x-ray to radiotherapy the tumor bed and surrounding areas after tumor resection. It is more convenient to carry out one-time targeted radiotherapy for the tumor bed. Two large phase III randomized controlled IORT trials, ELIOT and TARGIT-A, showed that the IBTR risk of IORT after breast-conserving surgery was slightly higher than that of WBI. In ELIOT trial, IBTR rate was 4.4% after IORT and 0.4% after WBI, but there was no difference in OS rate at 5 years (96.8% vs.96.9%). IORT significantly reduced the incidence of skin side effects such as erythema, dryness and hyper-pigmentation (*P* =0.0002) (68). In the TARGIT-A trial, the LR was 3.3% after low-energy x-ray IORT, and the LR of WBI was 1.3%. In subsequent analysis, LR rates of IORT and WBI were 2.1% and 1.1% (*P* =0.31) when immediate radiotherapy without pathological examination. The LR rates of IORT and WBI with delayed radiotherapy after pathological examination were 5.4% and 1.7%(*P* =0.069), respectively. Immediate IORT reduced the risk of local recurrence compared to delayed IORT (69). Long-term follow-up of the ELIOT trial showed that the incidence of IBTR was significantly higher in patients who received APBI than in the WBI group. The incidence of IBTR at 10 years was 8.1% in the ELIOT group and 1.1% in the WBI group, and the incidence of IBTR at 15 years was 12.6% and 2.4%, respectively (HR 4.6; *P* < 0.001). There was no difference in OS rates (ELIOT vs. WBI:83.4% vs. 82.4%), and the increased risk of IBTR did not shorten the OS of patients. The results indicated that patients who choose electron beam IORT should be at low risk for IBTR (70). The median follow-up of TARGIT -A was 8.6 years, and the longest follow-up was 18.9 years. The results showed that TARGIT- IORT was not-inferior to WBI in the local control. The local relapse-free survival, OS, breast cancer mortality, and the risk of non-breast cancer death were of no significant difference. The risk of non-breast cancer death was significantly reduced by 41% in patients in the TARGIT- IORT (HR 0.59, *P* =0.005). This trial showed that risk adapted single dose targeted radiotherapy during lumpectomy can effectively replace WBI after breast cancer surgery (71).

Noninvasive image-guided breast brachytherapy (NIBB) is a new APBI approach using the AccuBoost brachytherapy system. The system consists of a pair of breast immobilization plates, a kilovoltage x-ray tube, a film cassette and a series of specialized applicators. The patient’s breast was placed between a pair of the plates and a kilovoltage image was obtained for identification and localization of the tumor bed. Then a pair of treatment probes of the appropriate shape and size were selected to enclose the target volume and radiation was delivered using a high-dose-rate Ir-192 source via remote afterloader (72). In a prospective phase II trial, 28.5Gy/5F, once-daily NIBB was used to treat patients with early breast cancer after breast-conserving surgery. All patients were well tolerated, with a median follow-up of 14 months, and no late toxicity or local recurrence were observed (73). Another prospective study found similarly good safety in 40 breast cancer patients treated with NIBB once daily at 34Gy/10F. No grade≥3 late toxicity were observed. The incidence of grade 2 toxicity at 2 and 5 years was 5% and 10%, respectively. The good cosmetic effect at 5 years reached 100%. The 5-year IBTR was only 6.7%, and 5-year OS was 93.7% (74). NIBB had a low incidence of late toxicity and good cosmetic appearance, which needs to be verified by a larger cohort.

Magnetic resonance image-guided radiotherapy (MRIgRT) has advantages of providing superior contrast images of soft tissue, real-time imaging, no additional radiation dose, and the ability to use imaging markers. The MR-Linac system can obtain changes in the biological characteristics of patient’s specific tumor and normal tissue during radiotherapy and adjust it according to the treatment response of the patient to achieve real-time anatomical and biological MRI-guided adaptive radiotherapy to provide highly personalized radiotherapy (75, 76). MR-guided stereotactic APBI (MRgS-APBI) can effectively protect more normal tissues and reduce the number of irradiation segmentation. In two prospective trials, MRgS-APBI was successfully delivered to 48 breast cancer patients who received a single dose of 20Gy and 19 who received three doses of 8.5Gy. Two clinical trials in all patients achieved 88.5% of dosimetry goals during the period, and no technical problems related to MRgS-APBI that caused the patient to stop treatment occurred. Those evidence proved the feasibility of MR-guided stereotactic body radiotherapy (SBRT) APBI (77). MR-Linac has opened up a new pattern of image-guided radiotherapy and has been gradually applied to clinical practice. However, the current effect electron steam effect (ESE) generated during the treatment will affect the dose distribution. For breast cancer patients, the electron steam scattered into the air under the magnetic field will drift a long distance along the main axis of the magnetic field, up to the patients chin area above and down to the patients’ abdomen. ESE significantly changes the dose distribution in the field of radiation. The study found that the dose D_1cm3_ of breast cancer patients in the chin skin area corresponding to 1cm^3^ volume was 454.6cGy, which could reach 9.09% of the prescribed dose (5000cGy). After the addition of 1cm protective film, the dose of D_1cm3_ in chin skin decreased to 113.6cGy, which was similar to that without magnetic field (78). The first study of PBI patients treated with unity1.5T magnetic resonance accelerator found that the maximum dose (2.6Gy) of ESE induced chin skin under magnetic field reached 6.5% of the prescribed dose (40.05Gy). After adding 1cm bolus, the dose of chin skin could be effectively reduced to only 0.72Gy (79). Therefore, before radiotherapy of breast cancer on MR-Linac system, the possible impact of ESE should be pre evaluated, and certain measures should be taken to reduce the generation of low-dose areas. MRIgRT is the most advanced image-guided radiotherapy equipment in the world, which can achieve real precision radiotherapy. However, the clinical data of tumor patients treated with this technology are limited, and we still need to continue to explore in the future to provide more evidence for clinical practice.

Whether APBI can be used effectively is directly related to greater convenience and saving of medical resources. At present, the local control rate and cosmetic effect of APBI are still controversial. The best segmentation method and implementation technology of APBI still need a lot of trials to verify (**Table 4**). The update of APBI guidelines in 2016 set new standards for patients suitable for APBI. APBI is still a feasible choice for breast conserving patients who have been strictly screened (80).

**Table 4:** Clinical trials of accelerated partial breast irradiation in this review.

Authors (reference)	Number of patients	APBI techniques	Fractionation schedule TTD/fractions	Median F/U (years)	Results
Strnad et al. (60)	1184	Multicatheter brachytherapy	APBI:32.0Gy/8 fractions or 30.3Gy/7 fractions or HDR brachytherapy(50Gy with pulses of 0.60-0.80Gy/h, one pulse per h 24h/day) WBI:50.4-50.4Gy/25-28 fractions/5 weeks	6.6	5-year cumulative incidence of LR: APBI VS. WBI (1.44% VS. 0.92% *P*=0.42) The 5-year risk of grade 2–3 late side-effects to the skin: APBI VS. WBI (3.2% VS. 5.7% *P*=0.08) The risk of severe (grade 3) fibrosis at 5 years: APBI VS. WBI (0% VS. 0.2% *P*=0.46)
Coles et al. (61)	2018	Standard EBRT(standard tangential fields)	Whole breast group: 40Gy/15 fractions Reduced dose group: WBI 36Gy plus tumor-bed 40Gy/15 fractions; Partial breast group: local 40Gy radiotherapy/15 fractions	72.2 months	5-year cumulative incidence of local relapse: Whole breast(1.1% HR 1), Reduced dose(0.2% HR 0.33), Partial breast(0.5% HR 0.88)
Olivotto et al. (62, 63)	2135	3D-CRT	APBI:38.5Gy/10 fractions WBI:42.5Gy/16 fractions or 50Gy/25 fractions	8.6	8-year IBTR rate: APBI VS. WBI(3.0% VS. 2.8% HR 1.27) The increased rate of 3-year adverse cosmesis: APBI VS. WBI(29% VS. 17% *P*<0.001).
Vicini et al. (64)	4216	3D-CRT brachytherapy	APBI:3D-CRT 38.5Gy/10 fractions Brachytherapy 34Gy WBI:50Gy/25 fractions	10.2	10-year IBTR rate: APBI VS. WBI(4.6% VS. 3.9% HR 1.29)
Livi et al. (65, 66)	520	IMRT	APBI:30Gy/5 fractions WBI:50Gy/25 fractions	10.7	5,10-year IBTR rate: APBI VS. WBI 1.5% VS. 1.4%, HR 1.16; *P*=0.86; 3.7% VS. 2.5% HR 1.56; *P*=0.40 5,10-year OS rate: APBI VS. WBI 99.4% VS. 96.6% HR 0.17; P=0.057; 91.9% in both arms (HR 0.95; *P*=0.86)
Veronesi et al. (68, 70)	1305	IORT	IORT:21Gy/1 fraction WBI: 50Gy/25 fractions	12.4	In the IORT group: 5,10,15-year IBTR rate was 4.2%, 8.1%, 12.6%. 5,10,15-year OS rate was 96.8%,90.7%, 83.4%. In the WBI group: 5,10,15-year IBTR rate was 0.5%,1.1%,2.4%. 5,10,15-year OS rate was 96.8%, 92.7%, 82.4%.
Vaidya et al. (69, 71)	2298	IORT	IORT: 20Gy/1 fraction	8.6	The risk of non-breast cancer death was significantly reduced by 41% in patients in the IORT (HR 0.59, *P* =0.005).
Hepel et al. (74)	40	NIBB	34Gy/10 fractions	68 months	The actuarial 5-year IBTR was 6.7% and OS was 93.7%.

APBI, accelerated partial breast irradiation; TTD, total tumor dose; F/U, follow-up; HDR, high-dose-rate; WBI, whole breast; LR, local recurrence; HR, hazard ratio; EBRT, external beam radiotherapy; 3D-CRT, three dimensional conformal radiotherapy; IBTR, ipsilateral breast-tumor recurrence; IMRT, intensity-modulated radiotherapy; OS, overall survival; IORT, intraoperative radiotherapy.

## 5 Conclusion

In the era of “precision medicine”, radiotherapy should achieve the precision in three ways: accurate planning, accurate positioning and accurate treatment, killing tumor tissues as much as possible and minimizing exposure to normal tissues and organs. Therefore, with the continuous development of radiation therapy technology and the improvement of long-term survival rate, it is especially important to reduce the adverse effects caused by radiation therapy. For individual patients, according to the diversity of anatomy, the tradeoff between target coverage and the risk of secondary cancer is made to select the optimal treatment techniques. Short-range radiotherapy can not only shorten the treatment time, reduce the treatment cost, improve the quality of life of patients, but also make the utilization of medical resources more appropriate. In terms of clinical therapeutic effect, the local control rate, survival rate and adverse reactions of hypofractionated whole breast irradiation are no less than or even better than conventional fractionation radiotherapy. In light of this, it is expected to become the main treatment plan of postoperative radiotherapy for breast cancer patients. Based on current trials, APBI is still controversial in terms of local control rate and cosmetic effect. For specific patients with low-risk breast cancer after breast-conserving surgery, APBI is a safe and effective treatment, but it is still not considered to be functionally equivalent to whole breast radiotherapy. More clinical studies of APBI are needed to address the existing problems.

## Author contributions

JS and ZW drafted the paper, and contributed to writing and to critical review of the manuscript. All authors contributed to the article and approved the submitted version.
